# Supplementary material for: Association of Sarcopenia and Gut Microbiota Composition in Older Patients with Advanced Chronic Kidney Disease, Investigation of the Interactions with Uremic Toxins, Inflammation and Oxidative Stress
Source: Toxins (Basel). 2021 Jul 8;13(7):472. doi: 10.3390/toxins13070472 (PMC8309956; doi:10.3390/toxins13070472)
Supplement: Supplementary file 1 [file toxins-13-00472-s001.zip › toxins-1229090-supplementary.pdf]

Supplementary Materials: Association of Sarcopenia and Gut Microbiota Composition in Older Patients with Advanced Chronic Kidney Disease, Investigation of the Interactions with Uremic Toxins, Inflammation and Oxidative Stress

Elisabetta Margiotta, Lara Caldiroli, Maria Luisa Callegari, Francesco Miragoli, Francesca Zanoni, Silvia Armelloni, Vittoria Rizzo, Piergiorgio Messa and Simone Vettoretti

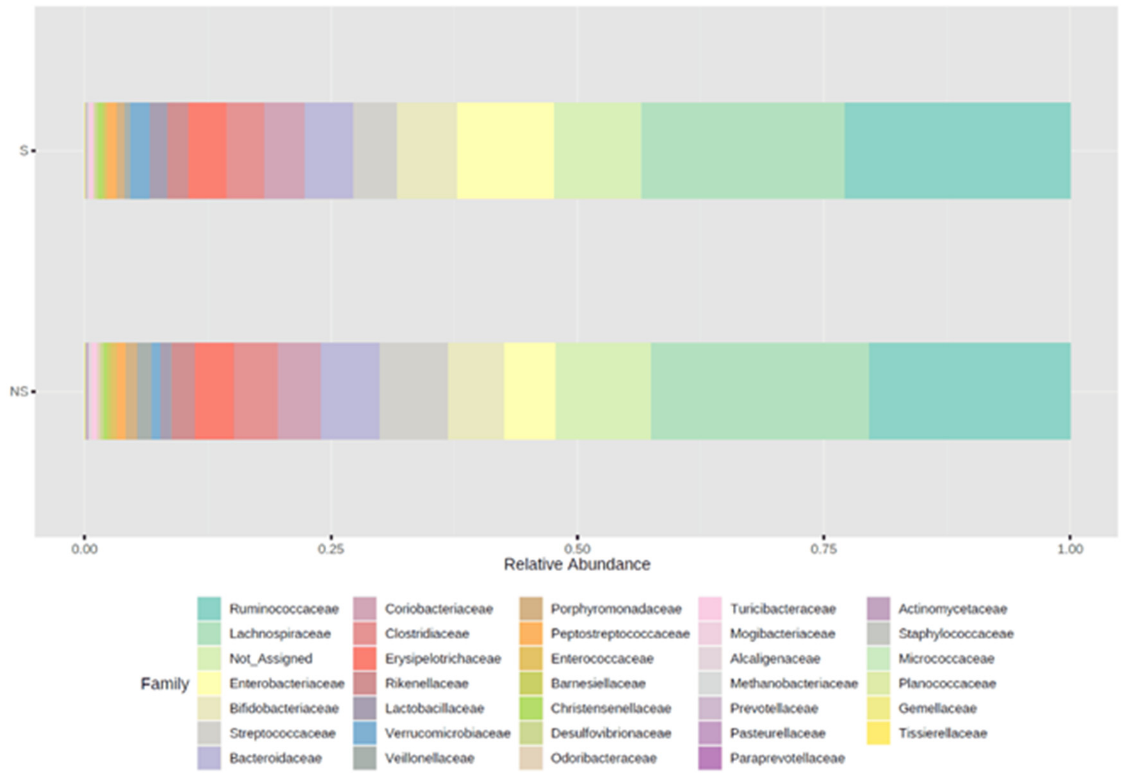

Figure S1: Distribution of bacterial families, expressed as relative abundance, in samples of sarcopenic (S) and not-sarcopenic (NS) subjects.

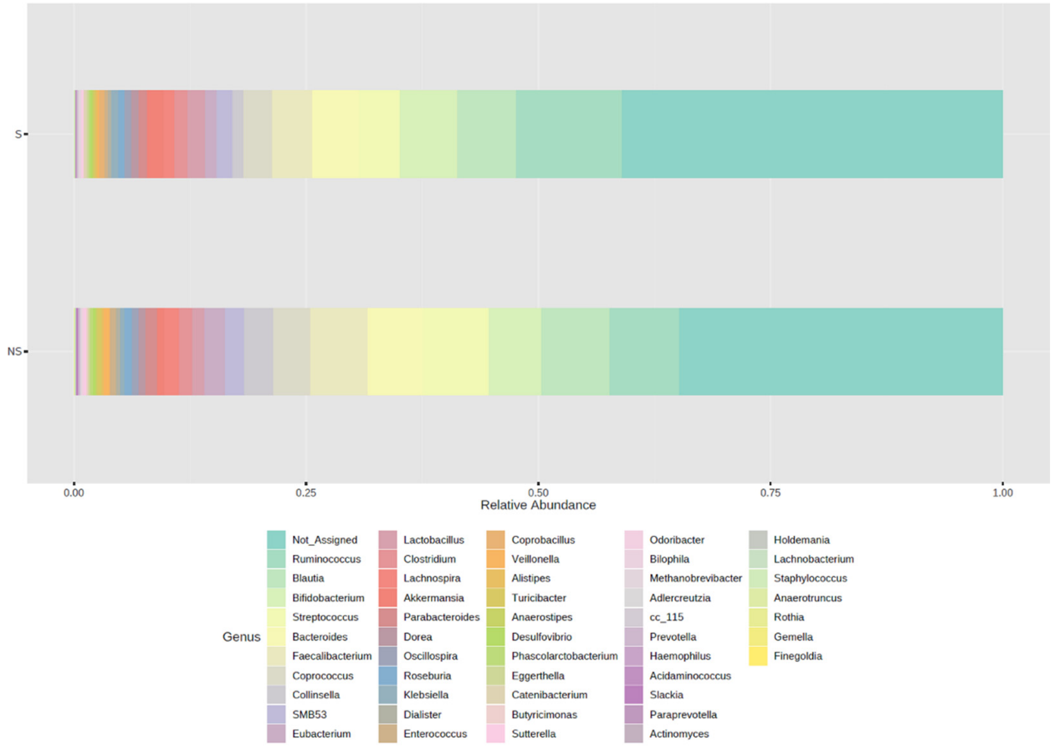

Figure S2. Distribution of bacterial genera, expressed as relative abundance, in samples of sarcopenic (S) and not sarcopenic (NS) subjects.

Table S1. Correlation between bacterial familiae and clinical and biochemical variables.

Bacterial familiae		**Sarcopenia**	**Age (yrs)**	**BMI (kg/m^2)**	**eGFR (ml/m**	**CRP (mg/dl)**	**MIS**	**diabetes**	**IS (umol/l)**	**PCs (umol**	**IL-10**	**Fetuin**	**IL-12p70**	**TNF alpha**	**IL-6**	**IL-17**	**MCP-1**	**MDA umol/l**
Actinomycetaceae	r	-0,062	0,001	0,069	0,092	-0,148	0,055	-0,027	-0,030	0,175	0,106	0,045	-0,398	0,096	0,149	0,288	-0,137	-0,108
	P	0,633	0,995	0,594	0,478	0,251	0,673	0,837	0,817	0,181	0,412	0,735	0,001	0,460	0,248	0,027	0,289	0,408
Alcaligenaceae	r	0,032	-0,133	-0,033	-0,068	0,162	0,144	0,164	0,142	0,128	-0,096	0,061	-0,062	0,128	0,206	0,100	-0,076	0,039
	p	0,806	0,306	0,798	0,601	0,209	0,266	0,202	0,279	0,331	0,458	0,646	0,638	0,320	0,108	0,452	0,557	0,767
Bacteroidaceae	r	0,034	-0,031	-0,196	-0,090	0,102	0,023	0,161	-0,004	-0,019	-0,150	-0,099	0,014	0,287	0,286	0,023	-0,156	0,046
	p	0,795	0,813	0,127	0,485	0,430	0,857	0,212	0,974	0,888	0,245	0,454	0,917	0,024	0,024	0,865	0,226	0,724
Barnesiellaceae	r	-0,194	-0,020	-0,162	-0,060	0,000	0,068	-0,037	-0,103	-0,074	-0,043	-0,122	0,144	0,216	0,090	-0,068	-0,141	0,081
	p	0,132	0,881	0,210	0,642	0,997	0,600	0,773	0,431	0,572	0,742	0,358	0,268	0,092	0,486	0,611	0,275	0,537
Bifidobacteriaceae	r	0,069	-0,170	0,138	0,162	0,093	0,094	0,024	-0,019	-0,166	0,175	0,114	0,097	0,050	0,018	-0,067	0,072	-0,160
	p	0,591	0,190	0,285	0,209	0,472	0,470	0,855	0,888	0,204	0,173	0,390	0,459	0,700	0,892	0,616	0,578	0,218
Christensenellaceae	r	0,090	-0,009	-0,287	-0,278	0,004	-0,030	-0,211	-0,052	0,264	-0,047	-0,091	0,065	0,048	0,026	0,021	0,060	0,063
	p	0,485	0,946	0,024	0,029	0,977	0,816	0,100	0,695	0,042	0,716	0,495	0,618	0,710	0,844	0,877	0,644	0,631
Clostridiaceae	r	0,044	-0,218	0,049	-0,271	0,031	-0,042	-0,159	0,163	0,233	0,227	-0,018	0,049	0,036	0,203	-0,165	0,153	0,034
	p	0,736	0,092	0,706	0,033	0,810	0,745	0,217	0,214	0,073	0,076	0,894	0,710	0,780	0,114	0,212	0,236	0,793
Coriobacteriaceae	r	-0,012	-0,068	0,003	0,159	0,245	0,164	0,037	-0,001	0,054	0,098	0,085	0,206	-0,098	0,020	-0,105	0,099	-0,027
	p	0,927	0,601	0,981	0,218	0,055	0,203	0,778	0,995	0,681	0,448	0,521	0,111	0,447	0,876	0,431	0,446	0,834
Enterobacteriaceae	r	0,191	0,020	-0,156	-0,037	-0,062	0,064	-0,062	-0,007	0,012	-0,147	-0,170	-0,018	-0,026	-0,096	-0,036	0,091	0,300
	p	0,138	0,877	0,225	0,774	0,633	0,623	0,632	0,960	0,927	0,254	0,199	0,892	0,838	0,457	0,784	0,480	0,019
Enterococcaceae	r	-0,050	-0,002	0,106	0,205	0,191	0,180	0,204	0,203	-0,081	0,093	0,109	-0,190	-0,030	0,083	0,111	-0,143	-0,028
	p	0,702	0,987	0,412	0,110	0,136	0,162	0,112	0,120	0,536	0,471	0,411	0,142	0,819	0,522	0,401	0,266	0,829
Lactobacillaceae	r	-0,133	0,014	0,160	0,231	0,156	0,102	0,121	0,415	0,022	0,004	0,182	0,034	-0,155	0,041	-0,132	0,208	0,096
	p	0,303	0,915	0,213	0,071	0,225	0,429	0,351	0,001	0,868	0,978	0,169	0,797	0,230	0,752	0,320	0,104	0,464
Micrococcaceae	r	0,075	0,075	0,082	0,169	-0,092	0,055	0,097	0,014	-0,108	-0,133	-0,016	-0,052	-0,122	-0,278	-0,073	-0,215	-0,039
	P	0,564	0,568	0,528	0,190	0,477	0,674	0,455	0,914	0,413	0,304	0,905	0,688	0,347	0,029	0,581	0,094	0,767
Paraprevotellaceae	r	0,042	-0,092	0,060	0,056	0,161	0,222	0,176	-0,004	-0,016	0,128	0,056	0,018	0,088	0,364	-0,060	0,110	0,199
	p	0,744	0,480	0,641	0,664	0,212	0,083	0,172	0,975	0,904	0,320	0,673	0,889	0,499	0,004	0,652	0,395	0,124
Pasteurellaceae	r	0,099	0,090	0,123	-0,094	0,190	0,054	-0,124	0,006	-0,043	0,051	-0,333	0,106	-0,161	-0,077	-0,184	0,055	0,074
	p	0,442	0,493	0,790	0,468	0,138	0,679	0,335	0,965	0,746	0,693	0,010	0,418	0,211	0,553	0,163	0,670	0,573
Peptostreptococcaceae	r	0,076	-0,067	-0,001	-0,161	0,093	-0,026	-0,111	0,087	0,108	0,236	-0,099	-0,023	-0,120	0,033	-0,275	0,154	-0,096
	p	0,555	0,608	0,994	0,212	0,471	0,839	0,393	0,509	0,413	0,064	0,457	0,861	0,353	0,796	0,035	0,233	0,464
Porphyromonadaceae	r	0,009	-0,072	-0,231	-0,105	0,016	0,019	-0,016	0,049	0,122	-0,046	-0,187	0,126	0,071	0,184	0,006	-0,057	-0,019
	p	0,945	0,582	0,071	0,415	0,900	0,881	0,905	0,711	0,352	0,721	0,155	0,331	0,584	0,152	0,965	0,657	0,885
Prevotellaceae	r	-0,060	0,045	-0,088	-0,283	0,137	-0,054	-0,143	0,058	0,191	0,155	0,049	0,054	-0,152	-0,035	-0,164	0,289	-0,003
	p	0,645	0,731	0,496	0,026	0,288	0,675	0,269	0,658	0,143	0,229	0,710	0,677	0,238	0,786	0,214	0,023	0,979
Rikenellaceae	r	0,073	0,031	-0,344	-0,169	0,123	0,087	0,022	-0,028	0,121	-0,152	0,007	0,051	0,152	0,191	0,067	-0,015	0,151
	p	0,570	0,815	0,006	0,189	0,342	0,504	0,866	0,833	0,357	0,238	0,960	0,695	0,238	0,136	0,611	0,905	0,245
Staphylococcaceae	r	0,072	-0,115	0,051	0,110	-0,282	-0,089	0,109	0,181	0,164	-0,035	0,016	-0,091	-0,194	-0,182	0,114	-0,117	-0,207
	p	0,579	0,376	0,694	0,395	0,026	0,493	0,398	0,165	0,210	0,788	0,907	0,488	0,131	0,156	0,391	0,363	0,109
Streptococcaceae	r	-0,008	-0,021	-0,027	0,229	-0,126	-0,028	0,241	0,141	-0,007	0,059	0,192	-0,179	-0,049	0,022	0,066	-0,168	-0,296
	p	0,951	0,871	0,837	0,073	0,328	0,828	0,059	0,283	0,959	0,650	0,145	0,168	0,706	0,864	0,621	0,193	0,021
Turicibacteraceae	r	-0,080	-0,034	-0,048	-0,178	0,017	-0,102	-0,235	-0,041	-0,100	0,037	0,062	0,151	0,048	-0,012	-0,197	0,097	-0,102
	p	0,534	0,794	0,711	0,167	0,893	0,430	0,066	0,754	0,447	0,773	0,642	0,247	0,714	0,925	0,135	0,452	0,434
Veillonellaceae	r	-0,208	-0,138	0,259	0,184	0,093	0,058	-0,033	-0,028	-0,213	0,124	-0,047	-0,054	-0,064	0,088	-0,010	-0,071	-0,007
	p	0,104	0,289	0,042	0,153	0,470	0,656	0,800	0,834	0,102	0,339	0,724	0,677	0,623	0,498	0,942	0,585	0,955
Verrucomicrobiaceae	r	0,230	0,069	-0,291	-0,085	-0,271	0,032	0,005	-0,074	0,152	-0,069	-0,008	-0,041	-0,084	-0,098	-0,045	0,029	0,117
	p	0,072	0,598	0,022	0,513	0,033	0,807	0,972	0,575	0,247	0,592	0,950	0,755	0,518	0,447	0,734	0,825	0,370

BMI, Body Mass Index; eGFR, estimated Glomerular Filtration rate; PCs, p-cresyl sulfate; IS, indoxyl sulfate; CRP, C-reactive protein; IL, interleukin; TNFα, Tumor nercosis factor alpha; MCP-1, Monocyte Chemoattractant Protein-1; MDA, malondialdehyde.

Table S2. Correlation between genus and clinical and biochemical variables.

Genus		Sarcopenia	Age (yrs)	BMI (kg/m^2)	eGFR (ml	CRP (mg/	MIS	diabetes	IS (umol/l)	PCs (umol/l)	IL-10	Fetuin	IL-12p70	TNF alpha	IL-6	IL-17	MCP-1	MDA umol/l
Actinomyces	r	-0,062	0,001	0,069	0,092	-0,148	0,055	-0,027	-0,030	0,175	0,106	0,045	-0,398	0,096	0,149	0,288	-0,137	-0,108
	p	0,633	0,995	0,594	0,478	0,251	0,673	0,837	0,817	0,181	0,412	0,735	0,001	0,460	0,248	0,027	0,289	0,408
Akkermansia	r	0,230	0,069	-0,291	-0,085	-0,271	0,032	0,005	-0,074	0,152	-0,069	-0,008	-0,041	-0,084	-0,098	-0,045	0,029	0,117
	p	0,072	0,598	0,022	0,513	0,033	0,807	0,972	0,575	0,247	0,592	0,950	0,755	0,518	0,447	0,734	0,825	0,370
Alistipes	r	0,139	0,159	-0,160	-0,054	0,113	0,155	0,274	-0,018	0,095	-0,065	0,036	-0,096	0,082	-0,014	-0,025	-0,060	0,086
	p	0,281	0,220	0,215	0,680	0,383	0,230	0,031	0,888	0,472	0,614	0,785	0,464	0,527	0,913	0,852	0,643	0,511
Blautia	r	0,004	0,019	0,178	0,040	-0,118	-0,063	0,035	-0,091	-0,258	-0,112	-0,111	-0,012	-0,072	-0,098	0,141	-0,126	-0,075
	p	0,976	0,885	0,166	0,759	0,363	0,628	0,789	0,487	0,047	0,385	0,401	0,930	0,578	0,446	0,288	0,328	0,567
Catenibacterium	r	-0,152	0,109	0,018	-0,189	0,034	-0,130	-0,237	0,111	0,279	0,063	-0,134	0,104	-0,044	-0,185	-0,197	0,235	0,004
	p	0,238	0,404	0,888	0,142	0,795	0,315	0,064	0,400	0,031	0,627	0,313	0,424	0,735	0,150	0,135	0,065	0,974
Clostridium	r	0,062	-0,094	0,069	-0,199	-0,121	-0,097	-0,105	0,081	0,089	0,201	-0,112	-0,080	-0,021	0,090	-0,239	0,172	-0,037
	p	0,635	0,473	0,596	0,122	0,348	0,454	0,417	0,537	0,501	0,118	0,397	0,540	0,869	0,488	0,069	0,182	0,778
Collinsella	r	-0,141	-0,123	0,067	0,199	0,198	0,052	0,051	0,044	-0,031	0,159	-0,003	0,209	-0,108	-0,006	-0,181	0,144	0,056
	p	0,274	0,346	0,604	0,121	0,123	0,690	0,693	0,737	0,813	0,216	0,980	0,106	0,406	0,966	0,171	0,265	0,670
Coprobacillus	r	0,108	-0,107	0,008	0,129	0,033	0,297	0,123	0,092	-0,057	0,161	0,189	0,060	-0,162	-0,048	0,046	-0,050	-0,043
	p	0,402	0,413	0,954	0,319	0,799	0,019	0,340	0,485	0,664	0,211	0,152	0,648	0,209	0,710	0,729	0,698	0,743
Desulfovibrio	r	-0,024	0,003	0,069	-0,036	-0,165	-0,223	-0,061	0,193	0,139	0,173	0,015	0,095	-0,150	-0,118	-0,066	0,263	0,124
	p	0,854	0,984	0,594	0,781	0,200	0,081	0,637	0,140	0,291	0,178	0,909	0,468	0,244	0,359	0,617	0,039	0,340
Dialister	r	0,017	-0,124	0,050	-0,090	0,067	0,079	-0,033	-0,166	-0,039	0,047	-0,259	0,054	0,064	0,063	-0,149	0,084	-0,004
	p	0,896	0,339	0,697	0,488	0,605	0,540	0,800	0,205	0,770	0,718	0,048	0,680	0,621	0,629	0,260	0,516	0,975
Dorea	r	-0,089	0,105	-0,004	-0,172	-0,037	0,226	0,104	0,215	0,288	0,028	-0,067	0,026	-0,082	0,061	-0,286	0,248	-0,076
	p	0,490	0,421	0,978	0,182	0,773	0,078	0,421	0,098	0,026	0,832	0,614	0,844	0,528	0,635	0,028	0,052	0,559
Eggerthella	r	0,177	0,219	0,011	-0,017	0,185	0,261	0,159	-0,060	-0,125	-0,190	0,011	-0,091	0,172	0,101	0,174	-0,188	-0,035
	p	0,169	0,090	0,935	0,898	0,151	0,040	0,217	0,651	0,341	0,140	0,932	0,488	0,183	0,437	0,188	0,144	0,788
Enterococcus	r	-0,050	-0,002	0,106	0,205	0,191	0,180	0,204	0,203	-0,081	0,093	0,109	-0,190	-0,030	0,083	0,111	-0,143	-0,028
	p	0,702	0,987	0,412	0,110	0,136	0,162	0,112	0,120	0,536	0,471	0,411	0,142	0,819	0,522	0,401	0,266	0,829
Faecalibacterium	r	-0,026	-0,224	0,190	-0,134	0,055	-0,065	-0,099	-0,003	-0,064	0,032	-0,087	0,162	0,045	0,060	-0,003	0,030	0,125
	p	0,842	0,083	0,139	0,299	0,674	0,617	0,446	0,984	0,627	0,805	0,512	0,213	0,726	0,641	0,980	0,818	0,338
Lachnobacterium	r	-0,078	0,098	0,091	-0,032	0,152	0,139	0,039	-0,198	-0,203	-0,002	-0,204	0,116	0,092	0,041	-0,014	-0,073	-0,013
	p	0,549	0,454	0,482	0,803	0,237	0,281	0,761	0,130	0,120	0,985	0,121	0,373	0,477	0,751	0,914	0,575	0,921
Lactobacillus	r	-0,133	0,014	0,160	0,231	0,156	0,102	0,121	0,415	0,022	0,004	0,182	0,034	-0,155	0,041	-0,132	0,208	0,096
	p	0,303	0,915	0,213	0,071	0,225	0,429	0,351	0,001	0,868	0,978	0,169	0,797	0,230	0,752	0,320	0,104	0,464
Oscillospira	r	0,004	-0,019	-0,214	-0,071	-0,004	0,184	0,226	0,016	0,223	0,075	0,045	-0,052	-0,113	0,071	0,042	0,073	-0,021
	p	0,976	0,885	0,095	0,585	0,974	0,152	0,077	0,904	0,087	0,560	0,736	0,693	0,380	0,585	0,752	0,571	0,870
Paraprevotella	r	0,042	-0,092	0,060	0,056	0,161	0,222	0,176	-0,004	-0,016	0,128	0,056	0,018	0,088	0,364	-0,060	0,110	0,199
	p	0,744	0,480	0,641	0,664	0,212	0,083	0,172	0,975	0,904	0,320	0,673	0,889	0,499	0,004	0,652	0,395	0,124
Prevotella	r	-0,060	0,045	-0,088	-0,283	0,137	-0,054	-0,143	0,058	0,191	0,155	0,049	0,054	-0,152	-0,035	-0,164	0,289	-0,003
	p	0,645	0,731	0,496	0,026	0,288	0,675	0,269	0,658	0,143	0,229	0,710	0,677	0,238	0,786	0,214	0,023	0,979
Ruminococcus	r	0,091	-0,133	-0,149	-0,143	-0,081	-0,075	0,106	-0,076	0,196	0,023	-0,002	-0,252	-0,008	0,063	-0,031	-0,227	0,078
	p	0,480	0,305	0,247	0,269	0,531	0,562	0,413	0,562	0,133	0,858	0,985	0,050	0,949	0,625	0,814	0,076	0,552
SMB53	r	-0,016	-0,248	0,164	-0,215	0,054	-0,078	-0,089	0,131	0,145	0,253	-0,111	0,049	-0,063	0,129	-0,209	0,146	0,091
	p	0,902	0,054	0,203	0,093	0,679	0,548	0,489	0,318	0,268	0,048	0,403	0,707	0,624	0,317	0,111	0,259	0,487
Streptococcus	r	-0,008	-0,021	-0,027	0,229	-0,126	-0,028	0,241	0,141	-0,007	0,059	0,192	-0,179	-0,049	0,022	0,066	-0,168	-0,296
	p	0,951	0,871	0,837	0,073	0,328	0,828	0,059	0,283	0,959	0,650	0,145	0,168	0,706	0,864	0,621	0,193	0,021
Sutterella	r	0,032	-0,133	-0,033	-0,068	0,162	0,144	0,164	0,142	0,128	-0,096	0,061	-0,062	0,128	0,206	0,100	-0,076	0,039
	p	0,806	0,306	0,798	0,601	0,209	0,266	0,202	0,279	0,331	0,458	0,646	0,638	0,320	0,108	0,452	0,557	0,767
Turicibacter	r	-0,080	-0,034	-0,048	-0,178	0,017	-0,102	-0,235	-0,041	-0,100	0,037	0,062	0,151	0,048	-0,012	-0,197	0,097	-0,102
	p	0,534	0,794	0,711	0,167	0,893	0,430	0,066	0,754	0,447	0,773	0,642	0,247	0,714	0,925	0,135	0,452	0,434

BMI, Body Mass Index; eGFR, estimated Glomerular Filtration rate; PCs, p-cresyl sulfate; IS, indoxyl sulfate; CRP, C-reactive protein; IL, interleukin; TNFα, Tumor nercosis factor alpha; MCP-1, Monocyte Chemoattractant Protein-1; MDA, malondialdehyde.
